# Essay on the Physiognomy of Serpents

**Published:** 1844-04-01

**Authors:** 


### Essay on the Physiognomy of Serpents. By *H. Schlegel.* Translated by *Thomas Traill,* M.D.

Notwithstanding the singularity of the title, the reader will find this a very valuable and interesting work. With regard to the use of the term *physiognomy*, the author explains it by stating that, instead of attempting to distinguish individuals by some isolated characters, he has endeavoured to present a faithful portrait of each species, considered in its different relations to allied species, to indicate the passage of one imaginary group to another, and thus to reduce the science to its most simple objects.

The characteristics of ophidians, according to M. Schlegel, consist in a very elongated body, furnished with a tail, and covered by a defensive armour of hard scales, which moves, supported by its ribs, by means of lateral undulations; the form is concentrated transversely into the smallest possible dimensions, but the parts are susceptible of great enlargement, so as to permit serpents to swallow the large animals intended by nature for their sustenance. For this purpose, the bony case of the head, instead of forming an immoveable mass, is composed of parts which, with the exception of those enclosing the brain, are susceptible of considerable motion, and usually in different directions. This is particularly the case with the lower jaw, the two branches of which are only united by elastic ligament. The total want of feet implies the absence of certain solid parts, as the sternum, pelvis, &c.; the ribs are free and enjoy an uniform mobility, contributing to the enlargement of the intestinal cavity, and to the changes of form which the trunk undergoes in the various movements of the body. The general integuments are divided into numerous compartments, forming so many articulations; the scales which form these articulations on the lower surface, are usually larger than the rest, and perform the office of feet: the ribs are attached to the lateral margin of the internal face of these plates. Between the scales there is a considerable amount of loose skin, to allow for the extraordinary enlargement of the internal parts. The whole structure, it will be seen, is especially adapted to the mode of locomotion and the manner of swallowing the prey.

*Of the Teeth and Poison-Gland.*—As ophidians swallow the animals on which they live whole, the teeth are not intended for mastication, but serve to detain the prey, to inflict wounds, and, frequently, to convey into the wounds which they make, a fluid secreted by glands lodged in the head. The glands are of two kinds; the one, salivary, is possessed by all serpents; the other, or poison-gland, is the property of venomous serpents only. The poison-gland is inclosed in a dense tendinous covering and attached posteriorly to the articulation of the lower jaw, anteriorly it ends in a pretty wide duct, which runs along the maxillary bone, and terminates in the orifice seen at the base of the fang. The teeth which conduct the poison into the wound are called fangs, they are hollow, and are always placed at the anterior end of the maxillary bone; they are concealed in the gum which here forms a sort of sheath, and are recumbent when the snake is in a state of repose, but are elevated when he intends to bite. The rest of the teeth, and all those of non-venomous serpents, are solid, with the exception of the cavity for the pulp. Some of the non-venomous serpents it is true have channels in the teeth, situated at the posterior part of the maxillary bone, but these are only for the purpose of pouring into the wound the saliva secreted by the posterior salivary glands. The fangs being more exposed than the other teeth, we find that Nature has placed behind them several germs of new fangs, sometimes as many as six, and in every degree of development; it is not known whether the old fangs are shed spontaneously at certain periods.

*Of the Poison.*—This, in its fresh state, is a transparent limpid fluid, greenish-yellow, slightly glutinous; when dried it becomes viscid and adheres firmly to substances; when heated it evaporates without inflaming; it is diffusible in water, which it renders turbid and whitish. Its properties have considerable affinity to those of mucus; it is neither acid nor alkaline; it has no peculiar smell; applied to the tongue, it produces the same sensation as grease. According to Fontana, it may be taken internally without inconvenience; but this is denied by Dr. Hering, who, after taking some doses of the venom of the Crotalus mutus, felt the effects for eight days and more; these effects manifested themselves by pains in the larynx and other parts of the body, by a copious secretion of mucus from the nose and œsophagus, by a frequent diarrhœa with pains in the rectum, &c.; to these were joined other very remarkable effects, due, according to Dr. Hering, to the effects of the poison on the moral faculties.

It is probable, however, that the venom produces its effects only when mixed with the blood; these effects are the more serious in proportion as the quantity of the poison has been considerable, and has been introduced into a part abounding in blood-vessels. Hence the bite of a large species is more dangerous than that of the small, and a deep puncture, or one in a vein, is almost always mortal. Other circumstances must also be taken into consideration, such as the size of the animal bitten as compared to that of the serpent; thus, in Europe, man rarely perishes from the bite of the viper; it requires from three to four vipers to kill a horse or an ox, while a single bite is sufficient to kill in a short time one of the small mammifera; but, in tropical climates, a bite from a large venomous snake is generally fatal both in man and in other animals. Serpents also in biting several times, expend their venom, so that the last wounds are less dangerous than the first.

The poison of ophidians affects much less the white-blooded animals than the vertebrata. In most of the latter, the effects of the bite manifest themselves immediately. Man speedily feels acute pain in the part where two minute punctures may be seen from which a few drops of blood flow; the wounded part swells, and inflammation occurs with more or less rapidity; the absorption of the poison is announced by general debility, walking becomes painful, respiration is impeded and laborious; the patient experiences burning thirst; nausea and vomiting quickly succeed, often followed by great distress and faintings, which, joined to the most violent pain, deprive the sufferer of his intellectual faculties. Livid spots surrounding the wound are the precursors of gangrene, which spreads to other parts of the body, and causes death after a longer or shorter interval. Even those who recover are sometims affected with partial or complete palsy of the affected parts, or even experience a continual disturbance of their intellectual faculties.

*Treatment of Snake-bites.*—Many plants have been spoken of as *antidotes*, such as the Ophiorhiza, Mungos, Aristolochia Indica, Polygala Senega, &c., but no dependence can be placed on them. The first precaution to use, when one has been bitten by a venomous serpent, is to clean the bitten part, in order to prevent the poison adhering to the skin from entering the scarifications, which our author recommends should be made immediately; the part may either be cut out, or destroyed by caustic, and the wound sucked, or what is better, the cupping-glass may be employed, if this is not at hand, a ligature may be applied round the limb, as near as possible to the wound, and between this and the heart. Internally, powerful stimulants, such as the *eau de luce*, may be administered. M. Lenz recommends chlorine, both externally and internally; arsenic also is much recommended, especially by Mr. Ireland, who employed it with great success in the West Indies. Frictions with olive oil have proved efficacious in some instances.

*Form of Serpents.*—This depends in great measure on their mode of life, the nature of the places, or the element they inhabit, and also on the kind of locomotion which is natural to them. Those which frequent trees are especially distinguished by their slender forms, while those which prefer plains, or retire into burrows, are recognizable by their compact body, terminated by a very short tail: intermediate between these two tribes as to development of parts, are a great many serpents which usually remain on the ground, but are able to climb and swim also with more or less facility; others that delight more in humid places, or never quit the water, present the most varied forms, more or less suited to this species of locomotion. The shape and disposition of the parts of the head give to each species a peculiar physiognomy which may serve for the recognition of the different races. The circumstances which chiefly contribute to render the physiognomy of serpents characteristic, are a large broad head, high, angular, cordiform, and covered with small scales with unequal surfaces, a wide mouth curved at its margins, thick lips, large fossettes on the sides of a muzzle truncated or turned up at the end, small eyes with an elongated pupil, and overhung by salient superciliary plates —characters which are generally united in the species with clumsy forms, such as we see in the serpents properly named venomous, and in some others. These pronounced features, however, do not always constitute the distinctive characters of dangerous ophidians.

*Enemies of Serpents.*—The curse pronounced against the serpent has rendered these reptiles universally detested. Not only does man kill them, indifferent whether they be venomous or inoffensive, but certain mammifera are produced in all the countries of the globe, that pursue serpents with persevering keenness. With us, their chief enemies are the badger, the hedgehog, the weasel, the martin, and the polecat; in tropical climates they have to contend against the civet, the ichneumon, and other carnivora. Several birds wage on them a continual war, such especially is the serpent-eater of the Cape, mounted on its long stilt-like legs, as it would seem on purpose to render the bites of snakes ineffectual; in South America the laughing falcon and other birds of prey pursue them eagerly; the large storks of India destroy an immense number of serpents; in Europe, besides the storks, ravens, kites, and several buzzards prey upon them. In tropical seas, there exist sharks that devour with avidity the sea-serpents; and lastly, many ophidians make war on each other, not even sparing their own species.

*Propagation.*—In our climates, where serpents only produce young once a year, copulation takes place generally in the first fair days of April or May. It is, at least in our indigenous species, three or four months before the eggs are ready to be laid; during this interval a species of incubation is going on in the belly of the mother; for, on opening the eggs just after they are laid, we almost always find a fœtus more or less developed, sometimes even perfectly formed. In this latter case, the young are shut up in a third membrane, which they tear at the moment of birth to commence their independent existence. In other cases, the young being only imperfectly formed when the eggs are laid, require sometimes the space of a month more before the hatching is accomplished. On this depends the distinction which has been made between viviparous and oviparous serpents; a distinction, it will be seen, not altogether correct. The number of eggs deposited at a time varies considerably in the different species; in some amounting to not more than ten, whilst it is said that our ringed snake lays as many as forty.

*Development.*—The young, on leaving the egg, differ from their parents, besides their size, by a system of colouring more vivid and more contrasted, by the bluntness and roundness of the head, by the largeness of the eyes, and by the less perfect state of the epidermis and its appendages. Their teeth, however, resemble those of adults. It was long believed that the tail of the young was shorter, in proportion to the trunk, than in the adult, and that this member presented fewer subcaudal plates. This opinion is probably not correct, because the number of plates corresponds to the number of vertebræ, and it is very improbable that fresh vertebræ should be produced.

Shortly after birth, the young ophidians undergo their first moult. This process is repeated in our climate five times in the year, namely, every month from the end of April to the beginning of September; during hybernation they do not cast their skin. "In order to reject the old epidermis which begins to detach itself at the head and especially along the borders of the lips, the serpent passes itself through mosses, grasses, or heaths, and contrives, by means of slow and continued movements or frictions, to disengage gradually the exterior layer of the skin, which is already replaced below by a new epidermis. The spoils thus removed are found inverted from one end to the other, forming a sac with a reticulated surface more or less diaphanous, more wide than the body of the snake, because of the dilatations of the membranous intervals, and presenting, with the exception of those of the mouth and the nostrils, no other orifice than the anus; for it is well known that the hemispherical membrane that protects exteriorly the globe of the eye, is part of the integuments, and comes off along with the rejected skin." Moulting, it would appear, is produced in the same manner in all serpents.

We are altogether ignorant of the age attained by snakes, though it is probable that they live long, as do all other reptiles; we are equally ignorant whether they have a stated period of growth, or what may be its duration. In this climate it would appear that they arrive at puberty in the fourth year.

*Habitudes.*—Ophidians are to be found in every country where the conditions necessary to the existence of reptiles in general are to be found. Many snakes live together, and on friendly terms, such as most of the aquatic, and especially the sea-serpents, that shew themselves in immense shoals on the surface of the ocean. The venomous land snakes, on the contrary, generally lead a solitary life. Most ophidians choose their food indiscriminately from among the three first classes of vertebrate animals. Like other reptiles, serpents can do without food for a long time. Dr. Traill knew of two rattle-snakes living eighteen months without swallowing any food. We are ignorant whether snakes drink; no fluid has been found in the stomach on dissection. Incapable of supporting cold, which also deprives them of food, they retire on the approach of Winter into retreats secured against the inclemencies of the weather; in our climate and in North America, this occurs towards the month of October, and they re-appear about the end of March or April. In warm climates, however, they pass the whole year in a state of continual activity.

*Fables and Prejudices.*—The serpent performed a great part amongst the ancients, and appears as the representative of the *evil principle* in the mythology of most nations; Arimanes, assuming the form of a serpent, seeks in vain to overcome his antagonist Orosmandes, who represents the good principle in the idealism of the ancient Persians. Passing by, however, all the allegorical representations of the serpent, we will notice only a few of the fables of more modern times. Many travellers speak of serpents of a monstrous *size,* which they say they have encountered, and which they speak of as reaching to forty feet and upwards; to these they apply the name of Boa-constrictor, though the true Boa-constrictors are considerably inferior in size to other species of the Boa and the Python. We now know that the most gigantic do not surpass twenty to twenty-five feet in total length, and that their thickness is not above seven inches in diameter. In the first rank of all known serpents, in point of dimensions, stands the Boa Murina, a native of the equatorial regions of America. The Python Bivittatus, found in inter-tropical Africa and Asia, attains nearly the same size. In our climate, serpents are rarely more than five feet in length, but in middle Europe there is one species of Coluber which arrives at the length of eight feet.

We may well be astonished then at the accounts of Boas from forty to fifty feet long, that attack men, oxen, and tigers, and swallow them whole, after having covered them with a frothy saliva. Such tales are about on a par with that of the three sons of a colonist, successively dying at long intervals, of a wound caused by the fang of a rattle-snake remaining in the boot of their father, who had first died of the bite. The story of the Great Sea *Sarpent* is well known. Many other absurd accounts are noticed, most of them boasting an American parentage.

*Schlegel's Arrangement of Serpents.*—M. Schlegel divides serpents into innocuous and venomous. The INNOCUOUS consist of—

1. Burrowing Serpents.—Gen. I. *Tortrix.*
2. Vermiform Serpents.—Gen. I. *Calamaria.*
3. Terrestrial Serpents.—Gen. I. *Coronella.* II. *Xenodon.* III. *Hetorodon.* IV. *Lycodon.* V. *Coluber.* VI. *Herpetodryas.* VII. *Psammophis.*
4. Tree Snakes.—Gen. I. *Dendrophis.* II. *Dryiophis,* subdivided into A. *Dryiophis of the Ancient World;* B. *Dryiophis of the New World.* III. *Dipsas.*
5. Fresh Water Serpents.—Gen. I. *Tropidonotus.* II. *Homalopsis.*
6. Boaform Serpents.—Gen. I. *Boa.* II. *Python.* III. *Acrochordus.*

VENOMOUS SERPENTS.—These are distinguished by the existence of the *fang.*

1. Colubriform Venomous Serpents. (Inhabit the hot countries of both worlds; they are not found in Europe.)—Gen. I. *Elaps.* II. *Bungarus.* III. *Naja.*

2. Sea Serpents. (Found only in the inter-tropical latitudes of the Indian Seas, or of the great Pacific Ocean.)—Gen. I. *Hydrophis.*

3. Venomous Serpents, properly so called. (These are discovered in the five great divisions of the world.)—Gen. I. *Trigonocephalus.* (Not found in Europe nor in Africa.) II. *Crotalus.* (Peculiar to the New World; the tail is armed at the extremity either with a sounding instrument called a *rattle,* or with a hard sharp scale.) III. *Vipera.* (The only venomous snakes found in Europe.)

For the description of these varieties, as well as for an essay on the geographical distribution of ophidians illustrated by a chart, we must refer the reader to the work itself.

